# Mutational Screening of BRCA1/2 Genes as a Predictive Factor for Therapeutic Response in Epithelial Ovarian Cancer: A Consensus Guide from the Spanish Society of Pathology (SEAP-IAP) and the Spanish Society of Human Genetics (AEGH)

**DOI:** 10.1007/s00428-019-02709-3

**Published:** 2019-12-03

**Authors:** J. Palacios, M. de la Hoya, B. Bellosillo, I. de Juan, X. Matías-Guiu, C. Lázaro, S. Palanca, A. Osorio, F. Rojo, J.M. Rosa-Rosa, J.C. Cigudosa

**Affiliations:** 1 Servicio de Anatomía Patológica, Hospital Universitario Ramón y Cajal, 28034 Madrid, Spain; 2 Instituto Ramón y Cajal de Investigación Sanitaria, 28034 Madrid, Spain; 3 Universidad de Alcalá, 28801 Alcalá de Henares, Spain; 4 CIBER-ONC, Instituto de Salud Carlos III, 28029 Madrid, Spain; 5 Molecular Oncology Laboratory, Hospital Clinico San Carlos, IdISSC (Instituto de Investigación Sanitaria del Hospital Clínico San Carlos), Madrid, Spain; 6 Laboratorio de Diagnóstico Molecular, Servicio de Patología, Hospital del Mar, 08003 Barcelona, Spain; 7 Unidad de Biología Molecular, Servicio de Análisis Clínicos, Hospital Universitario y Politécnico La Fe, 46026 Valencia, Spain; 8 Servicio de Anatomía Patológica, Hospital Universitario de Bellvitge, 08908 L’Hospitalet, Spain; 9 Unidad de Diagnóstico Molecular, Institut Català d’Oncologia, (ICO-IDIBELL-ONCOBELL), 08908 L’Hospitalet, Spain; 10 Human Cancer Genetics Programme, Spanish National Cancer Centre (CNIO), 28029 Madrid, Spain; 11 CIBER-ER, Instituto de Salud Carlos III, 28029 Madrid, Spain; 12 Departamento de Patología, Fundación Jímenez-Díaz, 28040 Madrid, Spain; 13 NIMGenetics, Parque Científico de Madrid, Campus Cantoblanco, 28049 Madrid, Spain

**Keywords:** BRCA, Next-generation sequencing (NGS), Ovarian cancer (OC)

## Abstract

Germline/somatic *BRCA*-mutated ovarian carcinomas (OC) are associated to have better response with platinum-based chemotherapy and long-term prognosis than non-*BRCA*-associated OCs. In addition, these mutations are predictive factors to response to Poly(ADP-ribose) polymerase (PARP) inhibitors. Different positioning papers have addressed the clinical recommendations for *BRCA* testing in OC. This consensus guide represents a collection of technical recommendations to address the detection of *BRCA1/2* mutations in the molecular diagnostic testing strategy for OC. Under the coordination of Spanish Society of Pathology (SEAP-IAP) and the Spanish Society of Human Genetics (AEGH), these recommendations have been developed by pathologists and geneticists taking into account previously published recommendations and their experience in the molecular characterization of these genes. Since the implementation of *BRCA* testing as a predictive factor can initiate the workflow by testing germline mutations in the blood or by testing both germline and somatic mutations in tumor tissue, distinctive features of both strategies are discussed. Additionally, the recommendations included in this paper provide some references, quality parameters, and genomic tools aimed to standardize and facilitate the clinical genomic diagnosis of OC.

## Introduction

Ovarian cancer (OC), the most aggressive gynecologic malignancy, produced 42,704 deaths in Europe in 2012 (1). This high lethality can be attributed, among other factors, to its frequent late stage at presentation and limited treatment options. OC is a heterogeneous disease that includes five main histological types [53]: high-grade serous carcinoma (HGSC), low-grade serous carcinoma (LGSC), endometrioid carcinoma (EOC), clear cell carcinoma (CCC), and mucinous carcinoma (MC). These histological subtypes have different epidemiological and genetic risk factors, and they differ also with respect to precursor lesions, pattern of tumor spread, prognosis, and response to common therapy approaches, such as chemotherapy, hormone therapy or poly(ADP-ribose) polymerase (PARP) inhibitors (PARPi) [44]. For example, HGSC, representing 70% of late-stage tumors, is associated in some cases with a specific precursor lesion (serous intraepithelial carcinoma of the fallopian tube), and has a poor prognosis, even though approximately 50% of the patients show an initial good response to platinum therapy. From a molecular point of view, approximately 95% of HGSC carry *TP53* mutations and about 50% have homologous recombination deficiency (HRD) due to alterations in genes involved in the homologous recombination DNA repair pathway [63].

In the TCGA cohort, 20% of HGSC carried mutations in *BRCA* genes: 9% germline mutations in *BRCA1*, 8% germline mutations in *BRCA2*, and 3% somatic mutations affecting one of the two genes [63]. In addition, *BRCA1/2* mutations (both somatic and germline) have been reported in 15% and 10% of EOC and CCC, respectively [51].

Identification of *BRCA*-mutated OC patients is important for the following reasons:

- To identify *BRCA* germline mutation carriers (40% of patients with OC and pathogenic germline *BRCA1/2* variants have no family history of breast or ovarian cancer).
- Germline/somatic *BRCA*-mutated OCs are associated with better response to platinum-based chemotherapy (the standard of care in patients with late-stage OC) and long-term prognosis than non-*BRCA*-associated OCs.
- Germline/somatic *BRCA* mutation is a predictive factor to response to PARPi.

Current recommendations for BRCA testing vary among European countries. Thus, Vergote et al. [66] observed differences regarding testing criteria based on the histology of the tumors. Testing was offered for all ovarian cancers in the Netherlands, Italy, Scotland, and the Czech Republic, for all non-mucinous high-grade carcinomas in France and for all high-grade serous carcinomas in Germany, Belgium, and Portugal. Recently the ESMO-ESGO consensus conference recommended testing for *BRCA1/2* mutations for all patients with non-mucinous ovarian cancer [15].

In Spain, a national consensus issued by the Spanish Society of Pathology (SEAP-IAP) and the Spanish Society of Medical Oncology (SEOM) recommended that germline *BRCA1/2* mutation testing should be offered to all patients with high-grade non-mucinous ovarian carcinomas. In addition, somatic testing should be considered in cases negative for germline mutations. At present, although the consensus recognizes the potential role of testing other HRD genes, its clinical implementation is still low [49].

Provided that the clinical recommendations have been addressed by different positioning papers [31, 49, 66], our consensus guide represents a collection of technical recommendations to address the detection of *BRCA1/2* mutations in the molecular diagnostic testing strategy for OC. Under the coordination of Spanish Society of Pathology (SEAP-IAP) and the Spanish Society of Human Genetics (AEGH), these recommendations have been developed by pathologists and geneticists taking into account previously published recommendations [19] and their experience in the molecular characterization of these genes.

An important question regarding the implementation of *BRCA* testing as a predictive factor is whether to initiate the workflow by testing germline mutations in the blood or by testing both germline and somatic mutations in tumor tissue. Distinctive features of both strategies are summarized in Table 1. Additionally, the recommendations included in this paper provide some references, quality parameters, and genomic tools aimed to standardize and facilitate the clinical genomic diagnosis of OC.

**Table 1:** Characteristics to take into account of the BRCA testing on blood and tumor samples

	Blood BRCA testing	Tumor BRCA testing
Pre-analytical considerations	Pre-analytical conditions less critical	Risk of delayed fixation and overfixation
Analytical phase *NGS*	Detection of germline mutations	Detection of germline and somatic mutations
	Methods must detect at least ≥50% variant allele frequency	Methods must detect at least ≥5–10% variant allele frequency
	Recommended average sequencing coverage between 50 × –250× reads	Recommended average sequencing coverage between 500 × –2000× reads
	Possible to detect large deletions/rearrangements	Difficult to detect large deletions/rearrangements
	Unlikely to generate false-positive SNVs	Fixation artifacts/generation of false-positive SNVs
	Well-established validated NGS methods	Not fully available validated NGS methods
Post-analytical phase *NGS* (I)	Straightforward analysis	Complex analysis
	Simple and validated filter pipelines	More complex. Sensitive to filtering methods
	Heterozygous pathogenic variants (VAF = 50%)	Heterozygous pathogenic variants (VAF < 50%)
		False-negative results (VAF < 5–10%)
Post-analytical phase *NGS* (II)	Possibility to miss a group of patients (<10%) that could benefit for PARPi therapy	Identification all possible patients that can benefit for PARPi therapy based on *BRCA* testing
	Low percentage of VUS expected (<10% in a well characterized population)	Possibility of finding novel variants for which there is no information in databases and increase the % of VUS
		More information needs to be included in the report which makes it more laborious

### Pre-analytical Considerations

Any pre-analytical factor that modifies the quality of the sample could potentially impact in the results [29, 33]. A recent publication has reviewed pathology practices to ensure molecular integrity biospecimens for precision medicine [17]. The labeling, preparation, and formaldehyde fixation (if necessary) and the delivery to the laboratory in which the study is performed are the responsibilities of the professional in charge of obtaining the sample. The laboratory performing the molecular study must be able to handle different types of samples. The key recommendation in pre-analytical process in both tissue and blood samples are summarized in Table 2.

**Table 2:** Pre-analytical recommendations for *BRCA* testing

Sample type	Key recommendations
Tissue sample	Follow College of American Pathologists guidelines for fixation of tissue samples (Hammond 2010), 8–48 hr, depending on size of specimen
	Vacuum and controlled temperature
	Controlled parafinization protocols
	Large samples must be properly handle by a pathologist or well-trained personnel in order to assure formaldehyde penetration and adequate fixation
	Cytological samples also useful because alcoholic fixatives excellently preserve DNA
	Sample must contain a percentage of tumor cells that is at least 30%. Avoid inflammation, immune infiltrates, and necrosis
	Morphological diagnosis should always include the type and grade of the tumors in order to gain insight into genotypic/phenotypic correlations
	Decalcification reduces DNA yield and quality. If needed, avoid acid decalcification
Blood sample	EDTA or nucleic acids preserving agent tubes
	Sample processed immediately after its extraction (ideally in less than 30 min when tubes contain no preservatives)
	Tubes treated with heparin must be avoided, since it can inhibit PCR reaction
	Centrifugation of the blood sample must be performed at 4 °C using low speed (1000–2000 g for 10 min), to split the hematic pellet from the fractions corresponding to the buffy coat and the plasma
	Higher centrifugation speed and time should be avoided to prevent platelet and leucocyte lysis
	Fractions obtained after blood centrifugation can be stored at –20 °C during short periods of time and at –80 °C for long terms
	Leucocyte fraction, serum, plasma, and whole blood are acceptable for germline DNA (leucocytes preferred)

Briefly, in tissue samples, the cold ischemia time (Cit), the period of time since tissue is obtained and its fixation, impacts on DNA, RNA, and proteins in large surgery specimens, like ovary tumors [6]. To limit this effect, standard procedures in tissue preservation in fixative process or freezing in optimum cutting temperature (OCT) compound are recommended for optimum DNA recovery. However, DNA is more frequently isolated from formalin-fixed paraffin-embedded (FFPE) samples and is highly recommended that the tissue fixation procedure be controlled by the pathology laboratory [18, 59, 68] [57, 61] [22]. Finally, morphological diagnosis should always include the type and grade of the tumors in order to gain insight into genotypic/phenotypic correlations. Representative tumor area selection and percentage of malignant cells must be performed by a well-trained pathologist on an H&E section. A minimum of 30% of tumor cellularity is recommended to guarantee the detection of a variant through molecular techniques [21].

Blood samples should be collected in tubes with either EDTA or nucleic acids preserving agents, and processed immediately after its extraction, ideally in less than 30 min when tubes contain no preservatives [36] and following standard procedures. The leucocyte fraction is an excellent source for the study of germline DNA, being the standard nowadays. The serum contains DNA from leucocytes, in worse preservation conditions, and the plasma contains circulating free DNA. However, the study of germline mutations can be performed using also whole blood without fraction separation.

DNA extraction based on precipitation, magnetic beads, or silica-based membrane columns shows similar efficiency upon quality and concentration of the DNA obtained [42]. However, it is highly recommended that DNA extraction is standardized and performed using CE-IVD marking techniques in both manual and automatized processes. These processes must be performed strictly following the manufacturer’s guidelines and standardized procedures after a privative validation process prior to the clinical use [10].

Measures for assuring full traceability of the sample and its derived products to avoid cross-contamination are critical. For tissue samples, precautions such as the preparation of free nucleic acid plastic containers, the use of PCR quality water for floating baths, and the systematic cleaning of devices with DNA and RNA removers [20] should be taken.

The use of spectrophotometry (Nanodrop), fluorometry (Qubit), and/or PCR fragment (Agilent Bioanalyzer) systems is recommended for the quantification and qualification of the DNA obtained, especially in tissue sample where formaldehyde and paraffin treatments produce the fragmentation of the DNA [42]. Isolated DNA must be kept at –20 °C, while PCR products and library prepared should be kept in a different freezer at –20 or –80 °C to avoid cross-contamination. There is not a large amount of information about the impact of the storage time on the quality of the DNA or amplification products, but, in general, their stability is assumed for several years [43].

### Analytical Phase: *Next-Generation Sequencing (NGS)*

Library preparation adapts the nucleic acids to be sequenced, including the fragmentation of the DNA by either physical (sonication) or enzymatic (endonuclease cocktail or transposase-mediated fragmentation) methods, adapter linkage, and the enrichment of the regions of interest (ROI). There are several strategies for ROI enrichment, and differences should be taken into account prior to the sequencing process. Amplicon-based methods are characterized by shortened and simplified preparation protocols also requiring smaller DNA inputs and no fragmentation step. This is especially relevant when working with small FFPE clinical specimens. This technology provides higher on-target values, while hybridization capture-based methods demonstrated higher uniformity rate. However both approaches have demonstrated technical and clinical utility in diagnostic procedures [56].

Molecular barcodes are introduced to allow sample identification and combination in a unique sequencing reaction [32]. After library preparation, appropriated quality control steps should be performed to determine the feasibility of the sample to continue the procedures. These quality control steps include quantification of the library, size analysis of the fragments, and quantitative PCR using adapter to check the real amplifiability of the library fragments.

The combination of sequencing platforms and commercially available sequencing kits offers a variety of options for next-generation sequencing (NGS) studies [9, 30, 37]. However, it is important to acknowledge that cost efficiency and acceptable turnaround times will only be achieved by fully exploiting the capacity of the sequencing runs, so sequencing strategy should be chosen based on the laboratory NGS testing demand [58]. In this regard, the use of commercially available panels (see Table 3) for *BRCA1/2* testing is the most common praxis in clinical routine. Since they have been validated by the manufacturers, their implementation has turned out to be easier. *BRCA1/2* genes are included in several NGS panels, such as BRCA panels in which only these genes are tested, hereditary cancer panels including other cancer-predisposing genes, and somatic cancer panels designed to detect oncogenic mechanisms in FFPE samples.

**Table 3:** Comparative information about commercial kits available for *BRCA1/2* testing

Genes	NGS panel name	Manufacturer	Technology	BRCA 1/2 coverage	Samples compatible	Input amount	BRCA alterations	Applicatons	Marked CE-IVD
*BRCA1/2*	Oncomine BRCA Research Assay	ThermoFisher Scientific	Amplicon-based	All coding regions and flanking intronic regions	Peripheral blood and FFPE samples	20 ng	SNVs, indels, and CNV	Mutational screening of BRCA1/2 in germinal or somatic studies	No
	GeneRead QIAact BRCA 1/2 Panel	QIAGEN	Amplicon-based	All coding regions and flanking intronic regions	Peripheral blood and FFPE samples	40 ng	SNVs and indels	Mutational screening of BRCA1/2 variants in germinal or somatic studies	No
	CleanPlex BRCA1 & BRCA2 Panel	CleanPlex	Amplicon-based	All coding regions and flanking intronic regions	Peripheral blood and FFPE samples	10 ng	SNVs and indels	Mutational screening of BRCA1/2 variants in germinal or somatic studies	No
	BRCA MASTR Dx	Multiplicon	Amplicon-based	All coding regions	Peripheral blood	100 ng	SNVs and indels	Mutational screening of BRCA1/2 variants in peripheral blood or somatic studies	Yes
	BRCA Tumor MASTR Plus	Multiplicon	Amplicon-based	All coding regions	FFPE samples	80 ng	SNVs, indels, and CNVs	Mutational screening of BRCA1/2 variants in somatic studies	Yes
	BRCA MAQ	Multiplicon	Amplicon-based	All coding regions	Peripheral blood	20 ng	CNV	Copy number variant (CNV) detection in BRCA 1/2 genes	No
Hereditary cancer panel (<40 genes)	GeneRead QIAact BRCA Advanced DNA UMI Panel	QIAGEN	Amplicon-based	All coding regions and flanking intronic regions	Peripheral blood and FFPE samples	40 ng	SNVs, indels, and CNV	Hereditary cancer panel including BRCA1/2, TP53, and most of PTEN	No
	Hereditary Cancer Solution (HCS)	SOPHiA GENETICS	Hybridization	All coding regions and flanking intronic regions	Peripheral blood	200 ng	SNVs, indels, and CNV	Hereditary cancer panel including BRCA1/2 among 26 cancer-predisposing genes	Yes
	CleanPlex Hereditary Cancer Panel	CleanPlex	Amplicon-based	All coding regions	Peripheral blood	10 ng	SNVs and indels	Hereditary cancer panel including BRCA1/2 among 37 cancer-predisposing genes	No
Cancer panel (>40 genes)	Human Breast CancerGeneRead DNA seq	QIAGEN	Amplicon-based	All coding regions	Peripheral blood and FFPE samples	10 ng	SNVs and indels	Cancer panel of 44 genes associated to breast cancer	No
	CleanPlex OncoZoom Cancer Hotspot Panel	CleanPlex	Amplicon-based	Unknown	Peripheral blood and FFPE samples	10 ng	SNVs and indels	Cancer panel of 65 genes associated to solid tumors	No
	Oncomine Comprehensive Assay v3	ThermoFisher Scientific	Amplicon-based	All coding regions	FFPE samples	10 ng	SNVs and indels*	Cancer panel of 170 genes associated to solid tumors	No
	TruSight Tumor 170	Illumina	Hybridization	All coding regions	FFPE samples	40 ng	SNVs, indels, and CNV †	Cancer panel of 170 genes associated to solid tumors	No
	TruSight Oncology 500	Illumina	Hybridization	All coding regions	FFPE samples	40 ng	SNVs and indels ‡	Cancer panel of 523 genes associated to solid tumors	No

*Panel is able to detect SNVs and small indels in 134 genes, amplifications in 47, and fusion/splice variants in 51

†Panel is able to detect SNVs and small indels in 148 genes, amplifications in 59, and fusion/splice variants in 55

‡Panel is able to detect SNVs and small indels in 523 genes and fusion/splice variants in 55. Additionally includes tumor mutational burden and microsatellite instability determination

In conclusion, the choice of the NGS panel for library preparation will depend on the type of study (somatic, germinal, or both) and the sequencing strategy available in the institution [56]. A good NGS testing strategy of *BRCA1/2* genes should allow the identification of single nucleotide variants (SNVs) and small insertions-deletions (indels) in all coding exons and exon-intron boundaries, as well as CNVs, although the latest can be determined by other techniques such as multiplex ligation-dependent probe amplification (MLPA).

### Post-analytical Phase I: Analysis and Filtering of Variants

Data analysis is performed after the sequencing process and can be divided into three stages: primary, secondary, and tertiary analysis. The *primary analysis* consists of base calling and demultiplexing processes and is performed automatically by specific software on the sequencer. Base calling is the conversion of raw data to nucleotide reads, and the demultiplexing process separates data from each sample according to specific barcodes. A quality control of the run is also performed at this stage by the technical staff to verify the amount of the library pool loaded as well as the general sequencing process. Although there are different file formats to store primary analysis results, FASTQ and BAM are the most commonly used.

The *secondary analysis* can be done on the same sequencer or employing external pipelines or workflows [26]. Quality control of each sample reads; elimination of low-quality reads, adapters, primers, and duplicated reads; and alignment are the basic processes along this step. During the alignment step, each read is compared against the reference sequence of the human genome to locate its original position, which can be improved using the distance and orientation of *paired-end* reads [50]. The most widely used mapping file format is the Sequence Alignment Map (SAM) and its compressed version Binary Alignment Map (BAM) [55]. Depending on the enrichment technique used for library preparation, after the initial mapping, duplicate reads might be removed to avoid false-positive variant calls due to unwanted clonal amplification of reads with sequence artifacts. Duplicate reads can be eliminated based on the genomic position or because they share a UMI (unique molecular identifiers). The addition of UMIs before PCR amplification discriminates between alleles arising from the same genomic locus and sequencing reads produced by PCR amplification [13]. After alignment, genome browsers, like the Integrative Genomics Viewer (IGV), propose the majority or highest-quality base for each position [41]. Finally, reads can be recalibrated, and the indels realigned, by consensus, to the left [55]. *Variant calling* is the step where the variants (bases that differ from the reference genome) are identified for each sample. Variant calling format (VCF) is the standard file to store the information of variants (one line per variant). The variant information from different individuals can be stored in a single VCF file. Variant filtering criteria vary depending on the NGS platform and the enrichment system used, like the minimum number of reads covering the base, as well as the minimum average coverage and depth accepted for a variant. In the case of tumor samples, the variant allele frequency (VAF) varies in function of the tumoral cellularity present in the sample and the tumor proportion with the alteration [50] (the minimum average read depth to detect variants with a VAF of 5% should be 500X). An accurate filtering increases the quality of the results, e.g., filtering out reads with a quality value <Q30 can greatly improve the dataset, as quality score of 30 represents an error rate of 1 in 1000X [55] with a corresponding call accuracy of 99.9%. In *BRCA1/2* the most frequent variants observed are single nucleotide polymorphisms (SNPs), indels, single nucleotide variants (SNVs), and copy number variations (CNVs) [50]. One limitation of current NGS approaches is the ability to reliably detect CNVs, since NGS mainly depends on the panel employed and the quality of the DNA samples. In somatic test, the high heterogeneity increases the background noise in the CNV copy plot, leading to frequent false results. Several bioinformatics tools for CNV assessment have been developed [38].

The *tertiary analysis* consists of the functional annotation of the variants identified in the individuals and the selection of pathogenic variants (see post-analytical phase II section). In this stage, the technical and biological information is integrated to interpret the variants detected through the sequencing process [50, 55].

Data analysis is the most complex and time-consuming stage of the entire NGS process; therefore, qualified personnel are required, either a geneticist or a molecular biologist appropriately trained to handle the complex classification of genetic variants across BRCA genes. In case of somatic mutation analysis, the importance of the pathologist expertise is stressed, since the impact of pre-analytical conditions, selection of the appropriate tissue fragment, and interpretation of the results in the light of microscopical appearance are crucial, providing an integrated molecular pathology report. BRCA1/2 genes have a large size, and many clinically relevant variants have been located throughout the whole coding region of the genes that have to be entirely analyzed. The quality of the DNA from FFPE samples is typically low, and the tumor percentage is variable; thus the variants can be identified at allelic frequencies less than 50%, leading to a complex identification of the variants, compared to peripheral blood samples. For all these reasons, the accurate pipeline validation and the staff experience play an important role in the *BRCA1/2* genes analysis, which is even more crucial when tumor samples are studied.

### Post-analytical Phase II: Variant Interpretation

Clinical classification should be done following the guidelines developed by the American College of Medical Genetics and Genomics/Association for Molecular Pathology (ACMG/AMP) [5, 27, 54] and the (*evidence-based network for the interpretation of germline mutant alleles)* ENIGMA consortium [25] (an adaption of the ACMG/AMP rules to the specific case of *BRCA1/2*). The ACMG/AMP guidelines propose 28 classification criteria (frequency in control populations, co-segregation, functional data, and in silico predictions, among others), categorize them according to their strength for a benign or pathogenic assertion, and propose rules to combine different criteria into a final five-tier classification of variants that reflects their probabilities of being clinically relevant (see Table 4).

**Table 4:** IARC five-tier classification, adapted from [49]. *Based on the recommendations from the EMQN (European Molecular Genetics Quality Network)

Class	Probability (quantitative)	Description (qualitative)	Report?	Predictive testing?	Management of carriers	Research testing
5	>0.99	Definitely pathogenic	Yes	Yes	Full high-risk guidelines	No
4	0.95–0.99	Likely pathogenic	Yes	Yes	Full high-risk guidelines	May be helpful
3	0.05–0.949	Uncertain	Yes*	No	Variant irrelevant to risk assesment (Manage risk based on family history)	May be helpful
2	0.001–0.049	Likely not pathogenic	No*	No	Variant irrelevant to risk assesment (Manage risk based on family history)	May be helpful
1	<0.001	not Pathogenic	No*	No	Variant irrelevant to risk assesment (Manage risk based on family history)	No

Most genetic variants will be readily classified as *benign* based on allele frequency in control population. Indeed, an allele frequency ≥0.01 in any general continental population dataset of at least 2000-observed alleles is considered a benign stand-alone criterion for a *BRCA1/2* variant [5, 25]. There are many databases reporting frequencies [11, 23, 24, 47, 48] . One of the most widely used database to identify non-pathogenic variants is the Genome Aggregation Database (gnomAD) [11], which contains information about 125,748 exomes and 15,708 genomes from unrelated individuals. Other variants will be readily classified as *likely pathogenic*, based on its location in a canonical splice site, or *pathogenic*, based on the generation of a premature termination codon not located in the last exon of the gene (PTC-NMD).

We will focus here on the *variants of unknown significance* (VUS), which are typically missense or intronic variants. A high proportion of the former VUS in *BRCA1/2* has been classified, thanks to international efforts that have allowed compilation of evidences and standardization of classification rules [5, 25, 27, 28, 52, 54]. There are three reputable databases [1, 39, 46] that compile accurate information on *BRCA1/2* variants classification based on multiple evidences from different sources combined into a Bayesian model to generate a posterior probability and validated by an expert panel [28]: ClinVar, BRCA Exchange, and BRCA Share. In ClinVar, the classification is reliable only if the “review status” contains three stars, meaning that it has been validated by the ENIGMA [25] expert panel, while BRCA Exchange [1] provides the same classification as ClinVar in a more user-friendly environment and more detailed information [14], including a mobile app that provides reclassification updates on variants of interest. On the other hand, BRCA Share™ [7] compiles data from 16 academic laboratories performing *BRCA1*/*2* testing in France. It is important to highlight that other databases or levels of curation (e.g., <3 stars in ClinVar) might not be reliable. For that reason, variant interpretation requires expertise and should be performed by a specialist, avoiding automatic interpretations.

Most variants remaining of uncertain significance after filtering are rare missense, synonymous, or intronic variants (MAF < 0.01). If pathogenic, missense variants are often assumed to impact the protein structure/function, but the possibility of the underlying nucleotide change leading to aberrant splicing should not be disregarded [65]. Over 400 in silico tools have been developed to predict the functional impact of missense changes [2], including Align GVGD, PolyPhen, SIFT, and MutationTaster. None of these tools is gene specific, but due to the clinical relevance of *BRCA1/2* testing, Align GVGD has been calibrated to predict the probability for any missense change in these two genes to be pathogenic [54].

In silico tools predicting splicing alterations are far more accurate. MaxEntScan has been calibrated to predict the *prior* probability of any possible substitution in *BRCA1/2* to be pathogenic due to an impact on splicing [64]. Due to its accuracy, the ENIGMA expert panel has included a specific role for well-calibrated splicing tools in its classification process. More specifically, rare synonymous or intronic variants (IVS + 20_IVS–40 range) not predicted to affect or create splice sites are considered likely benign. By contrast, variants predicted alter splicing cannot be considered automatically pathogenic, distinguishing between spliceogenicity (the variant is altering splicing) and pathogenicity (the splicing alteration is pathogenic). For that reason, in the absence of RNA analysis, variants located in the acceptor site of *BRCA1* exons 8, 9, 10, 13, and 14 and *BRCA2* exon 12 and variants located in the donor site of *BRCA1* exons 9 and 10 and *BRCA2* exon 12 should be considered VUS. Align GVGD- and MaxEntScan-calibrated predictions are updated periodically [35] and have been incorporated as well into the BRCA Exchange detail view.

#### Splicing Studies

The most likely mechanism underlying the pathogenic nature (if any) of synonymous and intronic variants is aberrant splicing. A recent report by the ENIGMA consortium describes some aspects relevant to design, perform, and interpret adequately studies performed by RT-PCR in blood derived RNA from carriers. [67]. If the study demonstrates that the variant allele produces *only* PTC-NMD transcripts and/or transcripts lacking the coding sequence of critical functional domain, it can be classified as pathogenic. If RNA from carriers is not available, or if the splicing outcome observed does not permit a definitive classification, complementary studies performed by validated reporter minigenes might be useful.

### Reporting Germline and Somatic *BRCA1/2* Results

The laboratory must determine which variants, according to its clinical significance, are informed and how the results will be communicated. For *BRCA1/2* genes, there is a broad international consensus to use the five-classes classification [56]. All pathogenic and likely pathogenic variants (classes 5 and 4, respectively) must be informed; reporting of VUS (class 3) is recommended, with the statement that it should not be used in a clinical decision-making context.

The report should contain the identification data of the patient and his/her diagnosis, the name of the physician requesting the test, and the reason for referral. Methodology of the diagnostic test performed and its scope should be accurately described (see Table 5). Reports should contain a summary of the results clearly framed and a discussion of the clinical relevance and the limitations of the test. Reports must be signed by at least two qualified faculties who have reviewed, approved and interpreted the results and should clearly identify the laboratory and its contact information, as well as its accreditations-certifications and participation in quality assessments. The report must be clear and concise; one page is the preference for the European Molecular Quality Network (EMQN) [3] and the Genomics Quality Assessment (GenQA) [4]; and two are accepted when reporting NGS results to provide the information specified here (important content information is described in Table 5) [12, 45, 60] and other information that the laboratory wish to report should be provided as supplementary and clearly separated from the main report. The laboratory must agree with the clinical team and the delivery time of the results; although considering its relevance for certain therapeutic decisions, the laboratory should ensure a fast delivery time in cases that need it.

**Table 5:** Recommended content information for a proper report of *BRCA1/2* mutational test

Sample	• Description of the sample analyzed	Peripheral blood DNA, DNA from FFPE tissue
	• In the case of studies carried out on tumor samples, indicate the suitability of the sample	Neoplastic content estimation and cellularity, limit of detection of the test performed (the percentage of mutated allele that is detectable with the technique used, experimentally determined during the validation process)
Technique	• Description of the analytical determination	Horizontal extent of the region analyzed, sequencing platform and chemistry used, methodology of enrichment of the genes, steps of bioinformatics pipeline
	• Description of the NGS relevant quality parameters	Global depth of coverage, minimum coverage of all analyzed bases, percentage of bases with a coverage >30X (500X in somatic and must be accompanied by the limit of detection of allelic frequency)
	• Has the analysis of the number of copies of the genes been performed?	YES (by MLPA, NGS, others) or NO
	• Limitations of the test	Analytical sensitivity, detection of deep intronic variants or large indels, existence of areas of low coverage, the accurate sequencing of homopolymer regions (>8 nt)
	• Genes reference sequence number/version	Database version for annotation and HGVS (Human Genome Variation Society) nomenclature for cDNA and amino acid changes
Variants	• Definition of the criteria used to filter out variants	Information from databases supporting pathogenicity or the potential therapeutic implications
Recommendations	• Negative germline reports should advise a tumor study for the determination of somatic *BRCA1/2* mutations	
	• Positive *BRCA1/2* somatic reports should recommend a germline study to determine the nature of the mutation identified and its correct interpretation in the personal and family context of cancer	
	• Reports with positive *BRCA1/2* mutation should recommend the referral of the patient to a genetic counseling unit for the purpose of an adequate interpretation of the results and their personal and family implications	

### Recommendations and Quality Programs

In order to ensure a correct clinical practice, it is recommended that laboratories have certification and accreditation to perform molecular genetic studies. Certification is defined by ISO as the “Procedure by which a third party gives written assurance that a product, process or service conforms to specific requirements.” Certification is performed typically according to the ISO9001-2015 standard, and, although provides a quality measure, it does not ensure that the laboratory has demonstrate technical competence to produce valid data and results. The requirements of certification address the quality management system (QMS) and include procedures and a quality manual, document control, define non-conformities (NCs) and corrective and preventive actions (CAPA), perform internal audits, and enhance customer satisfaction, but it does not necessarily include requirements of technical or analytical competence.

Accreditation is defined by ISO as the “Procedure by which an authoritative body gives formal recognition that a body or person is competent to carry out specific tasks.” The standard developed by ISO most frequently used in medical laboratories is the ISO15189 and ensures technical competence of a laboratory to perform specific types of testing by complying with specific management and technical requirements. The accreditation process also considers the QMS, like certification, but it has additional formal requirements of technical competence, including initial and continuous training of personnel, validation of methods and instruments, as well as internal and external quality control [8]. As a result, certification (typically according to the ISO9001 standard) should not be interpreted to mean that a laboratory has demonstrated the technical competence to produce valid data and results.

There is also a difference in the body that carries out the assessment and delivers the certification or accreditation certificate. Laboratories applying for ISO 9001 certification will be audited by a certification body, a third party that is accredited by an accreditation body. Each country has multiple certification bodies, but there is only one recognized national accreditation body (NAB) in each country that assesses laboratories against internationally agreed standards (Regulation (EC) No 765/2008). In Spain this is *Entidad Nacional de Acreditación* (ENAC).

As part of the accreditation process, it is mandatory to provide internal quality controls (IQC) and to participate in external quality assessment (EQA). IQC is an internal verification that the test yields consistent results day after day and is defined as “the set of procedures undertaken by the staff of a laboratory for continuously assessing laboratory work and the emergent results, in order to decide whether they are reliable enough to be released” [69]. EQA implies testing of the same samples by different laboratories. This assessment may be done either by cross-validation of samples among laboratories or may be organized by external agencies. In the case of *BRCA1/2* assessment, this is covered by the quality programs of the EMQN and GENQA.

These programs evaluate not only the technical performance of producing analytical results from samples shared to all participating laboratories but also the accuracy of the report produced (data included from the patients, information regarding testing, accuracy of databases employed), as well as the interpretation of the genetic results. In the case of *BRCA* assessment, this is of utmost importance as mutational assessment may not only include the detection of pathogenic variants but also VUS or benign variants, which should be correctly identified. For this reason, in recent years, some quality control assessments have been only based in the interpretation of the *BRCA1/2* variants, without performing wet-lab testing.

### Beyond BRCA Testing

Germline or somatic mutations in HR genes other than *BRCA1/2* have been reported in approximately 10% of ovarian carcinomas, including both serous and non-serous histologies [51, 63]. In addition, it has been observed that mutations in other HR genes have a similar positive impact on OS and platinum responsiveness as *BRCA1/2* mutations [51]. Finally, HRD can be assessed by methods other than the analysis of mutations in HRD genes. As a consequence of HRD, typical genomic alterations, such as LOH, telomeric imbalance, and large-scale transitions, can be accumulated and can be measured. Ovarian carcinomas with high LOH (≥14–16%) determined by NGS showed good response to rucaparib [62]. In addition, LOH, together with telomeric allelic imbalance and large-scale transitions, generates an HRD score (MyChoice® HRD test, Myriad Genetics Inc., Salt Lake City, Utah), which when ≥42 is associated to benefit to olaparib [34, 40]. In accordance with these data, ESMO-ESGO consensus conference recommended that testing for mutations in other HR genes, in particular *RAD51C/D*, *BRIP1*, and *PALB2*, should be considered in patient with ovarian cancer. Current assays of HR function, although promising, cannot be used to exclude patients from PARP inhibitor therapy [16].
